# Treatment of Indolent Cutaneous B-Cell Lymphoma with Intralesional or Intravenous Rituximab

**DOI:** 10.3390/cancers14194787

**Published:** 2022-09-30

**Authors:** Christian Menzer, Adriana Rendon, Jessica C. Hassel

**Affiliations:** Division of DermatoOncology, Department of Dermatology, National Center for Tumor Diseases (NCT), University Hospital Heidelberg, Im Neuenheimer Feld 460, 69120 Heidelberg, Germany

**Keywords:** indolent cutaneous B-cell lymphoma (CBCL), primary cutaneous follicle center lymphoma (PCFCL), primary cutaneous marginal zone lymphoma (PCMZL), intralesional rituximab (ILR), intravenous rituximab (IVR), therapy recommendations

## Abstract

**Simple Summary:**

Cutaneous B-cell lymphomas (CBCL) are a heterogeneous group of mature B-cells neoplasms that present in the skin without evidence of nodal or systemic involvement. Despite being indolent in nature, they tend to recur in a third of patients after treatment. As repetitive treatments may be necessary for patients with CBCL, there is a need for gentle non-invasive therapy. Rituximab is a medication that targets CD20, a receptor regularly expressed on CBCL, and thereby destroys the cancerous cells. This medication can be given as an infusion into the vein or as an injection directly into the skin tumors. In this study, we found that rituximab injections have a similar efficacy compared to infusions for patients with limited CBCL lesions.

**Abstract:**

Indolent cutaneous B-cell lymphomas (CBCL) are a rare disease for which the therapeutic recommendations are based on clinical reports. Recommendations for solitary lesions include surgery or irradiation. However, the high relapse rates may require less invasive repeatable therapy. This study seeks to retrospectively assess the efficacy of intralesional rituximab (ILR) for indolent CBCL when compared with intravenous rituximab (IVR). Patients treated for indolent CBCL with ILR or IVR at the Division of DermatoOncology of the University Hospital Heidelberg were eligible for this study. Characteristics of lymphoma, treatment response, and adverse events were assessed. Twenty-one patients, 67% male at a median age of 52 (range 17–80), were included. Nineteen (90%) had only localized lymphoma (stage T1 and T2). Complete response was achieved in 92% (11/12) of ILR after a median of one cycle (three injections) and 78% (7/8) of IVR patients after a median of six cycles. Half of ILR patients and 78% of IVR patients showed relapse after a median of 15 and 23 months, respectively. Adverse reactions were usually mild and were limited to the first injection of ILR. One patient with IVR contracted a pulmonary infection. ILR may be an alternative to the intravenous administration of rituximab for localized indolent CBCL.

## 1. Introduction

Cutaneous B-cell lymphomas comprise approximately 25–30% of all cutaneous lymphomas, the majority being T-cell Non-Hodgkin lymphomas, such as Mycosis fungoides [1]. Fortunately, the entity of cutaneous B-cell lymphomas is mostly linked to a good prognosis in overall survival, once systemic involvement has been excluded. In 2008, the European Organization of Research and Treatment of Cancer (EORTC) and the International Society for Cutaneous Lymphomas (ISCL) revised the classification of primary cutaneous B-cell lymphomas, dividing them into three main groups. Thus, 83% of all B-cell lymphomas belong to the two indolent subtypes of primary cutaneous follicle center lymphoma (PCFCL) and primary cutaneous marginal zone lymphoma (PCMZL), with a five-year survival of 90–98% [2]. The third group of primary cutaneous diffuse large B-cell lymphoma, leg type/other (PCDLBCL, LT/O) shows an intermediate aggressive course with a five-year survival of 20–70%, and requires different treatment modalities [3].

As cutaneous B-cell lymphomas are a rare disease with an incidence of <1 per 100,000 patients/year, therapeutic recommendations mainly rely on retrospective studies based on case reports and case series [4]. There is a consensus that surgery and/or irradiation should be performed as first-line therapies in the case of well-demarcated circumscribed disease [2]. Various single reports about other therapies used, such as topical or intralesional glucocorticoids, interferon, or systemic antibiotics, can be found in the literature [3,5,6,7]. In more extensive cases, systemic application of the monoclonal chimeric CD20-antibody rituximab has been successfully used to treat indolent B-cell lymphomas [8]. Other reports support the local injection of rituximab to deplete malignant B-lymphocytes in recalcitrant disease [9].

The available data suggest that regardless of treatment, approximately a third of tumor lesions will recur, mostly at the same site as the initial lymphoma [10]. As surgery induces scarring and conventional radiotherapy may lead to pigmentation alteration, atrophy, teleangiectasis, and hair loss, they are not an optimal option for recurrent disease [11]. In contrast, intralesional rituximab has no destructive effect on perilesional tissue and can be re-administered multiple times in the case of relapse. Especially in candid anatomical sites such as the face, this feature makes it a favorable treatment option.

Therefore, in this retrospective study, we analyzed the outcome of intralesional (ILR) and i.v. rituximab (IVR) in patients with indolent cutaneous B-cell lymphoma treated at our center in order to further discuss the advantages and disadvantages of each form of therapy.

## 2. Materials and Methods

### 2.1. Subjects

To investigate the efficacy of treatments for indolent B-cell lymphoma, patient electronic medical records (EMR) at the Department of Dermatology/National Center for Tumor Diseases (NCT) of the University Hospital Heidelberg were examined. A database was created including patients with a history of indolent cutaneous B-cell lymphoma (PCFCL, PCMZL), who were treated at our center between July 2012 and May 2021. Of these patients (n = 49), only patients who received treatment with intralesional rituximab (ILR) or i.v. rituximab (IVR) when first presenting at our center were included (n = 21). If subjects had received prior treatment with ILR or IVR at other clinics, we counted whichever treatment was received first at our center. Information about the tumor characteristics and management were obtained by chart review. All patients had histologically confirmed PCFCL or PCMZL. The tumor node metastasis (TNM) stage was determined according to the TNM classification system for CBCL proposed by the International Society for Cutaneous Lymphoma (ISCL) and the Cutaneous Lymphoma Task Force of the EORTC [12]. The use of retrospective patient data for this study was in line with the ethical vote by the University of Heidelberg institutional review board (S-454/2015).

### 2.2. Treatments

This retrospective analysis focused on two different forms of treatment with CD20-antibody rituximab. Intralesional rituximab (ILR, MabThera), 50 mg/m^2^ body surface area (BSA) per cycle, was injected directly into each tumor on days 1, 3, and 5, followed by a 3-week pause. Injections were administered after topical anesthetic or without any premedication, depending on the patient’s preference. Acetaminophen of 500 mg up to six times daily was recommended for pain or flu-like symptoms after the injections, as needed. ILR was continued with further cycles if the tumor remnants were still clinically visible. Patients with intravenous rituximab (IVR, MabThera) received 375 mg/m² of BSA once weekly for 4 weeks, and were then re-evaluated for the prolongation of therapy with further cycles. Safety measure such as premedication with acetaminophen and antihistamines and a gradual increase in infusion rate were applied to reduce the risk for sensitivity reactions. In this study, patients were assigned to either an ILR or IVR treatment group, depending on which modality was first used when presenting at our center for the treatment of CBCL. Treatment efficacy and safety data including any adverse events during treatment with ILR or IVR were extracted from the chart reviews. Fluorescence-activated-cell-sorter (FACS)-based immunoassay reports were used where available to determine B-cell depletion after ILR or IVR. B-cell depletion was defined as peripheral CD19+ B-cells ≤ 0.1% of the total lymphocytes.

### 2.3. Statistics

All of the statistical analyses were performed using widely acknowledged statistical computer software (SPSS version 24.0; Chicago, IL, USA). The summary results are expressed as medians. Qualitative variables were expressed as percentages. Both therapy groups were compared for significant distributional differences using the Chi-square and Mann–Whitney-U test, respectively. Kaplan–Meier estimates of disease-free intervals and the presence or absence of disease relapse were compared using a log-rank test. Ninety-five % confidence intervals (CI) were calculated. The results were defined as significant at a *p*-value < 0.05.

### 2.4. Review Data

Two electronic databases (PubMed and Google Scholar) were used to search for current data in the literature. The electronic search included the following key words: “cutaneous b-cell lymphoma OR indolent lymphoma” AND “intralesional rituximab OR intravenous rituximab OR management”. The included articles comprised retrospective studies, case series, case reports, and reviews, with an emphasis on recent publications from the last five years.

## 3. Results

### 3.1. Subjects

Twenty-one patients were included in this retrospective analysis, of which twelve had ILR and nine had IVR for indolent CBCL after first presenting at our center. The median age was 52 years (range 17–80), and 67% were male and 33% female. 

Nineteen patients had only localized lymphoma (T1 and T2 in EORTC/ISCL classification 2008 [12]; 90%), whereas two patients had multilocal involvement (T3, 10%). The diagnosis of indolent lymphoma (PCFBCL or PCMZL) was histologically confirmed in all of the patients included in this study. CD20 expression had been detected in all patients with immunostaining before the initiation of therapy. The manifesting locations of the tumors were the head/neck area in 71%, trunk in 24%, and arms in 5%. Fourteen patients had primary cutaneous follicular B-cell lymphoma (PCFCL) (67%) and seven patients had primary cutaneous marginal zone lymphoma (PCMZL) (33%). Only 33% had not received any prior therapy in their medical history before the first cycle of rituximab at our center. Pretreatments, including those received at other oncological centers, were surgery (24%), ILR (24%), doxycycline (19%), irradiation (5%), or IVR (5%).

Twelve patients received treatment with ILR (57%) and nine patients received IVR (43%). Patients with multilocal disease (T3, n = 2) were all treated with IVR. A summary of the patient characteristics can be viewed in Table 1.

### 3.2. Treatment Efficacy

Patients were treated with a median of one cycle (one cycle = three injections; range 1–3 cycles) of ILR and six cycles of IVR (range of 6–11). Seven patients (33%) were treatment-naïve, while five (24%) had already received ILR and one had received (5%) IVR in the past.

**Figure 1:**
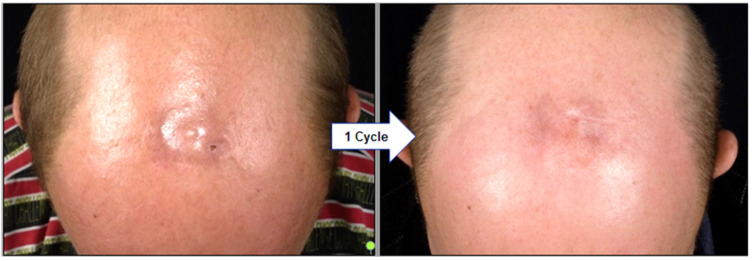
Clinical regression of PCFCL on the scalp in a 63-year-old patient three weeks after his first cycle of ILR. A median of one cycle (three injections) of ILR was required in our cohort to achieve complete remission (CR). PCFCL = primary cutaneous follicle center lymphoma; ILR = intralesional rituximab.

Complete clinical remission (CR) was obtained in 11 of 12 patients receiving ILR (90%), while one patient still had residual disease (partial response, PR) when treatment was stopped after two cycles. A median of one cycle (three injections) was needed to achieve CR status (range of 1–3 cycles) (Figure 1). Six patients (50%) showed a relapse of disease, all in the same anatomic location as the primary lesion. The median time to relapse in ILR patients was 14.9 months (range 6.7–56.8 months). Patients with a durable response (50%) had a median follow-up time of 49.9 months (range 4.4–92.7 months).

In the IVR group, four patients (44%) were treatment-naïve, while one (11%) had rituximab in the form of ILR in the past. CR was obtained in seven out of nine patients (78%) after a median of six infusions (range 6–11). However, 78% of IVR patients had a relapse after a median of 23.1 months (range 5.8–94.9 months). Patients with an ongoing response (22%) had a median follow-up time of 62.4 months (range 31.8–93.1 months).

**Figure 2:**
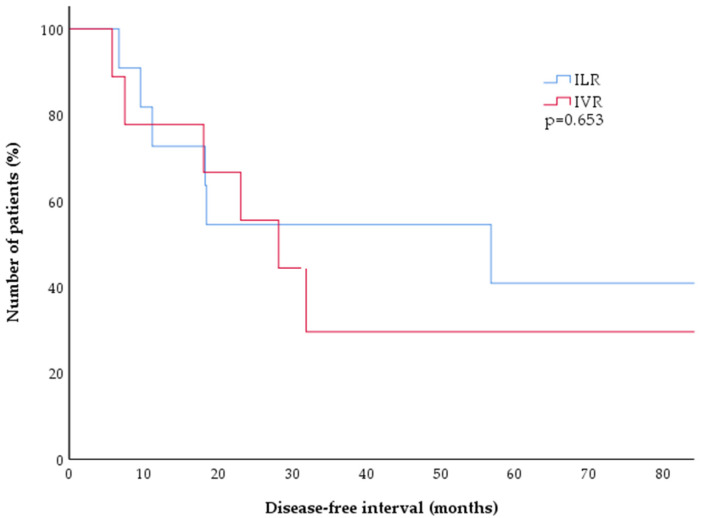
Kaplan–Meier estimates of disease-free survival after treatment of CBCL with either ILR (blue) or IVR (red). There was no significant difference between both treatment arms, with 50% of patients experiencing a relapse after approximately two years (*p* = 0.653). CBCL = cutaneous B-cell lymphoma; ILR = intralesional rituximab; IVR = intravenous rituximab.

All of the relapses in both cohorts were confirmed by biopsy. Histologic findings resembled the initial diagnostic lesion for each patient, with CD20 positivity confirmed in 11 of 13 cases and not done in the other two. There was no significant difference in the follow-up (F/U) times between the ILR and IVR group (*p* = 0.505), and no significant difference was found in the disease-free intervals between ILR and IVR treated patients (Kaplan–Meier estimate, Figure 2, *p* = 0.653).

### 3.3. Subgroup Analysis

There was equal distribution of previously treated and treatment-naïve patients in the ILR and IVR cohorts (*p* = 0.397). Untreated patients showed no significant difference in the frequency of relapse (*p* = 0.656) or in the duration of relapse-free times with either therapy (*p* = 0.3).

**Figure 3:**
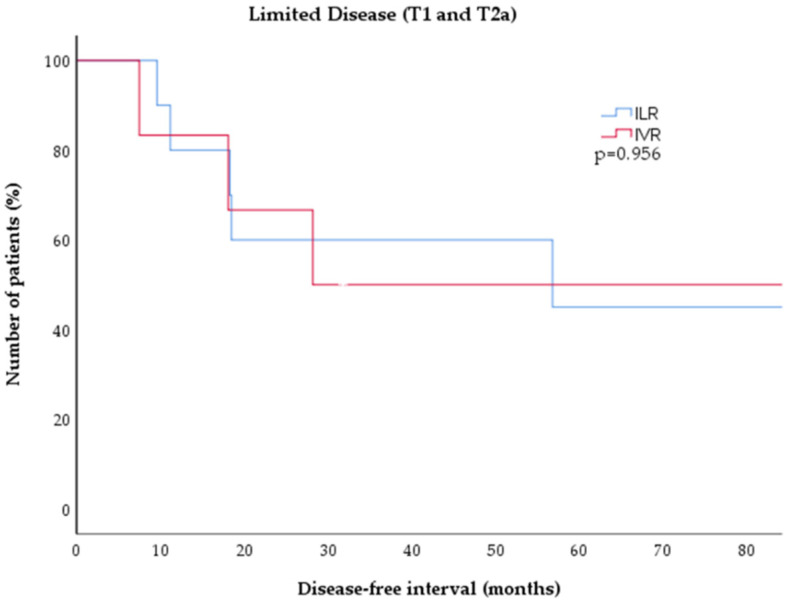
Kaplan–Meier estimates of disease-free survival in low-grade CBCL (T1 and T2 disease) after treatment with either ILR (blue) or IVR (red). There was no significant difference between tboth treatment cohorts (*p* = 0.826). CBCL = cutaneous B-cell lymphoma; ILR = intralesional rituximab; IVR = intravenous rituximab.

Seventeen patients (81%) had limited disease (T1a or T2a), of which 11 (65%) were treated with ILR and six were treated (35%) with IVR. The majority (16/17, 94%) achieved CR, while one patient (6%) showed PR after two cycles of ILR. Five patients (45%) with ILR experienced a relapse after a median of 18.3 months (range 9.6–56.8 months). Four patients (67%) with IVR had a relapse after a median of 23.2 months (7.5–94.9 months). There was no significant difference in the duration of disease-free intervals between both treatments for limited disease CBCL (*p* = 0.956, Figure 3).

There were four patients (4/21, 19%) with extensive disease (T2b, T2c, and T3b) in this study. One of these patients (T2b) was treated with ILR, showed CR after three cycles, and had a relapse after 7 months. Three patients with extensive disease (one with T2c and two with T3b) received IVR, where PR was achieved in one case (T2c) and CR in the other two cases (T3b), and had a relapse after 5.8 (T2c), 23.1 (T3b), and 31.9 months (T3b), respectively.

In the CBCL subgroup analysis, there were no statistically significant differences between PCMZL and PCFCL regarding distribution within the therapy cohorts (*p* = 0.35), frequency of response to therapy (*p* = 0.293), or duration of relapse-free intervals (*p* = 0.233). However, there was a significantly higher number of patients with PCFCL than PCMZL experiencing relapse (78.6% (11/14) vs. 29% (2/7), *p* = 0.026).

### 3.4. Safety Data

Seven patients (58%) treated with ILR complained of adverse reactions within hours after the first injection during the first cycle (Table 2). These consisted of headache, light fever, nausea, arthralgia, and fatigue, which were resolved within 24 h in all patients and did not recur with further injections.

In the IVR group, one patient (11%) developed a pulmonary infection which required i.v. antibiotics. Otherwise, there were no adverse reactions documented for IVR.

Fluorescence-activated-cell-sorter (FACS)-based immunoassay analyses from the peripheral blood were available for six patients after therapy with ILR (n = 3) or IVR (n = 3). The ILR patients showed post therapeutic B-cell decrease (0.3%) or full depletion (0%) in two out of three cases after one month, increasing to 2% and 4.1% after 7 and 10 months, respectively. The third ILR patient had a normal B-cell count in his only analysis at 11 months. In the IVR group, all patients showed post-therapeutic B-cell depletion (0%, n = 2) or decreased B-cell count (5%, n = 1) even after a median of 12 months (range 7–17 months) post treatment with IVR. All of them had normal B-cell counts in immunoassays before therapy.

## 4. Discussion

This retrospective study showed good results for the use of rituximab in indolent CBCL patients, not only for systemic, but also for intralesional application. The majority of patients experienced full clinical remission (CR) after a median of only one cycle of local injections with rituximab. No significant difference was seen in the therapy response rates between ILR and IVR. A relapse in 50% of our patients after a median time of 15 months was noted, which matched the relapse rates found in the literature. Senff et al. stated that approximately a third of patients with CBCL will experience a relapse of disease, regardless of treatment modality, which was also confirmed in a retrospective study with 52 patients by Olszewska-Szopa et al. [2,13]. Despite showing complete remission in most patients, relapse rates of 43% after surgery and 45% after radiotherapy within 5 years have been reported [2,14]. A similar relapse rate of 42% was confirmed for ILR in a comprehensive Spanish meta-analysis with a median follow-up time of 19 months (48 weeks) [9]. Although significantly more patients with PCFCL than PCMZL experienced a relapse in this study, this finding could not be confirmed by other studies in the literature, with varying relapse rates between 30–50% for both entities [13,15,16,17]. Thus, patients should be prepared for the possibility of chronically recurring disease.

The majority of patients in our study presented with tumors in the head and neck area, which is a precarious anatomical site in terms of disfigurement and cosmetic therapeutic outcome. In 92% of our patients, relapse tumors occurred in the same location as the previous lesions. The high risk of recurrence makes gentle treatments preferable over invasive and destructive approaches. Surgical excision bears the risk of scarring and disfigurement, while conventional radiation therapy (RT) may lead to irreversible alopecia, chronic dermatitis, and pigmentation alteration in approximately a fifth of patients [11]. RT has shown long-lasting remission without relapse at previously treated sites [2]. Low-dose involved-field RT may be an effective treatment for limited disease, especially in candid areas such as the head and neck, with an 86% response rate and a lower toxicity than conventional RT. Alternatively, ILR allows for non-scarring regression of lymphoma lesions and may be repeated in the case of relapse.

Complete response (CR) was achieved by most patients receiving rituximab as either IVR or ILR. Neither treatment showed a statistic advantage in comparison regarding relapse rates. Certainly, ILR is only feasible in limited disease when all lesions can be injected, while IVR may be a favorable option for extensive disease. Relapse rates for ILR of approximately 40–50% have previously been reported [17]. A retrospective analysis of 26 patients receiving IVR for CBCL with a median follow-up time of over 12 years showed relapse rates of 68% with IVR [18]. Thus, further research is warranted to assess whether locally directed therapy may be equally or even more effective in limited disease.

Over half of patients with ILR reported low-grade adverse reactions such as fatigue, headache, and light fever after their first injection. These symptoms resolved in all patients within 24 h and did not recur with subsequent injections. In the IVR group, only one patient had documentation of adverse events in the form of pneumonia after rituximab. As acute AEs were present in over half of patients with ILR, routine medication with acetaminophen or NSAIDs right before or shortly after injections may be advisable to prevent such reactions. Therapeutic regimens for IVR, including routine premedication with acetaminophen and antihistamines, may explain the lack of early onset AEs in the IVR cohort. IVR induces complete B-cell depletion within 72 h, and B-cell count normalization starts 6–9 months after therapy with normal levels after 9–12 months [19]. ILR has also been found to deplete peripheral CD20 and CD19 B lymphocytes for up to 6 months after injection [20]. In our study, two patients with ILR and all patients with IVR demonstrated B-cell concentrations below normal after therapy (e.g., <8%). Two patients post IVR even had persistent B-cell depletion (e.g., <0.1%) after 7 and 12 months, respectively. Severe adverse events under systemic rituximab are described as dose-dependent and include anaphylactic reactions, systemic inflammatory response syndrome (SIRS), and infections [21]. In particular, in the COVID-19 era, immunosuppression by complete B-cell depletion might be harmful for patients [22]. Intralesional injection may allow for the use of smaller dosages of rituximab, which may lower the occurrence of such severe adverse events. As an additional benefit, a reduced dosage may allow for lowering healthcare costs in the management of indolent CBCL. However, as shown in our data, systemic effects of rituximab may occur even after intralesional application. Thus, patients’ vaccination schemes should be controlled and updated before the initiation of either form of rituximab.

The advantages of ILR are its tolerability, with brief pain at injection sites, the need for minimal amounts to achieve a clinical response compared with the doses needed in systemic treatments, and the quick administration time [23]. Aside from frequent recurrence, indolent cutaneous B-cell lymphomas show a benign course of disease, with a normal life expectancy in 95–99% of patients [1]. Therefore, even an observational approach is among the recommendations for the treatment of indolent CBCL in the current National Comprehensive Cancer Network (NCCN) guidelines [24].

## 5. Conclusions

As indolent B-cell lymphomas are a rare disease and recommendations are based on case reports and case series, reassessment of the commonly used therapies is still of importance. In this retrospective analysis, ILR was proven to be a valuable therapeutic alternative to surgery, radiotherapy, and even IVR with regard to the response and relapse rates. Therefore, ILR may be a reasonable choice in the first-line management of lower tumor stages of indolent CBCL.

## Figures and Tables

**Table 1:** Patient characteristics of the included patients with indolent CBCL receiving either ILR or IVR.

CBCL Treatment	ILR	IVR	*p*-Value
**Patients total (n, %)**	**12 (100)**	**9 (100)**	
**Gender (n, %)**			0.061
Female	2 (17)	5 (56)	
Male	10 (83)	4 (44)	
**Age (median, range)**	**47 (17–60)**	**57 (39–80)**	0.075
**Indolent CBCL type (n, %)**			0.350
PCFCL	9 (75)	5 (56)	
PCMZL	3 (25)	4 (44)	
**Location (n, %)**			
Head–neck	8 (67)	7 (77.8)	
Upper back	1 (8)	2 (22.2)	
Chest	2 (17)	0 (0)	
Arms	1 (8)	0 (0)	
**TNM classification (n, %)**			
T1a	7 (58)	2 (22.2)	
T2a	4 (33)	4 (44.4)	
T2b	1 (8)	0 (0)	
T2c	0 (0)	1 (11.1)	
T3b	0 (0)	2 (22.2)	
**Limited disease (up to T2a)**	**11 (92)**	**6 (67)**	0.149
**Treatment naive (n, %)**	**3 (25)**	**4 (44)**	0.830
**Prior Therapy (n, %)**	**9 (75)**	**5 (56)**	0.350
Surgery	2 (17)	3 (33)	
ILR	4 (33)	1 (11)	
Doxycycline	2 (17)	2 (22)	
RT	1 (8)	0	
IVR	1 (8)	0	
Topical steroid	1 (8)	1 (11)	
**Best response (BR) (n, %)**			0.830
Complete response (CR)	11 (92)	7 (78)	
Partial response (PR)	1 (8)	2 (22)	
**Cycles to BR (median, range)**	**1 (1–3)**	**6 (6–11)**	
**Relapse (n, %)**	**6 (50)**	**7 (78)**	0.195
**Median time to relapse (months, range)**	**14.9 (6.7–56.8)**	**23.1 (5.8–94.9)**	0.568
**Relapse same location (n, %)**	**6 (100)**	**5 (71)**	
**Median F/U time (months, range)**	**50.0 (4.4–92.4)**	**62.4 (31.8–93.1)**	0.505

CBCL = cutaneous B-cell lymphoma; ILR = intralesional rituximab; IVR = intravenous rituximab; PCFCL = primary cutaneous follicle center lymphoma; PCMZL = primary cutaneous marginal zone lymphoma; RT= radiotherapy; F/U = follow-up.

**Table 2:** Safety data for the ILR and IVR cohorts. Over half of the patients with ILR experienced minor symptoms after their first injection without further AEs in the course of therapy. In the IVR cohort, no early onset AEs were reported. One patient (*) developed pneumonia 1.4 months after the sixth cycle of IVR. While the FACS analysis 7 months or more post therapy revealed reduced B-cell counts in 5 of 6 the available patients, only IVR-treated patients showed persistent full B-cell depletion (<0.1%) after such a long time.

Adverse Events (AEs)	ILR (n = 12)	IVR (n = 9)
**Any AEs, n (%)**	7 (58)	1 (11)
**Early onset AEs (after 1st application)**	6 (50)	0 (0)
Flu-like symptoms	6 (50)	0 (0)
Injection site pain	1 (8)	0 (0)
Nausea	2 (17)	0 (0)
**Late onset AEs**	0 (0)	1 (11) *
**FACS analysis (Peripheral Blood)**	**ILR (n = 3)**	**IVR (n = 3)**
CD19+ B lymphocytopenia (<8%, after ≥7 mo)	2 (67)	3 (100)
Complete B-cell depletion (<0.1%, after ≥7 mo)	0 (0)	2 (67)

AEs = adverse events; ILR = intralesional rituximab; IVR = intravenous rituximab; FACS = Fluorescence-activated cell sorter; mo = months.

## Data Availability

The data can be shared upon request.
